# *Helicobacter pylori* Impairs Murine Dendritic Cell Responses to Infection

**DOI:** 10.1371/journal.pone.0010844

**Published:** 2010-05-27

**Authors:** Ya-Hui Wang, Jean-Pierre Gorvel, Yen-Ting Chu, Jiunn-Jong Wu, Huan-Yao Lei

**Affiliations:** 1 Institute of Basic Medical Sciences, National Cheng Kung University, Tainan, Taiwan, Republic of China; 2 Aix Marseille Université, Faculté des Sciences de Luminy, Centre d'Immunologie de Marseille-Luminy (CIML), UMR6546, Marseille, France; 3 Inserm, U631, Marseille, France; 4 CNRS, UMR6102, Marseille, France; 5 Departments of Microbiology and Immunology, National Cheng Kung University, Tainan, Taiwan, Republic of China; 6 Medical Laboratory Science and Biotechnology, College of Medicine, National Cheng Kung University, Tainan, Taiwan, Republic of China; University of Hyderabad, India

## Abstract

**Background:**

*Helicobacter pylori*, a human pathogen associated with chronic gastritis, peptic ulcer and gastric malignancies, is generally viewed as an extracellular microorganism. Here, we show that *H. pylori* replicates in murine bone marrow derived-dendritic cells (BMDCs) within autophagosomes.

**Methodology/Principal Findings:**

A 10-fold increase of CFU is found between 2 h and 6 h p.i. in *H. pylori*-infected BMDCs. Autophagy is induced around the bacterium and participates at late time points of infection for the clearance of intracellular *H. pylori*. As a consequence of infection, LC3, LAMP1 and MHC class II molecules are retained within the *H. pylori*-containing vacuoles and export of MHC class II molecules to cell surface is blocked. However, formalin-fixed *H. pylori* still maintain this inhibitory activity in BMDC derived from wild type mice, but not in from either TLR4 or TLR2-deficient mice, suggesting the involvement of *H. pylori-*LPS in this process. TNF-alpha, IL-6 and IL-10 expression was also modulated upon infection showing a TLR2-specific dependent IL-10 secretion. No IL-12 was detected favoring the hypothesis of a down modulation of DC functions during *H. pylori* infection. Furthermore, antigen-specific T cells proliferation was also impaired upon infection.

**Conclusions/Significance:**

*H. pylori* can infect and replicate in BMDCs and thereby affects DC-mediated immune responses. The implication of this new finding is discussed for the biological life cycle of *H. pylori* in the host.

## Introduction

*Helicobacter pylori* is a Gram-negative, spiral-shaped, microaerophilic bacterium which colonizes the gastric mucosa. Infection occurs worldwide and is correlated with socioeconomic condition [1]. The prevalence among middle-aged adults is over 80 percent in many developing countries, as compared with 20 to 50 percent in industrialized countries. Overt diseases, however, occur in only 10–20% of infected individuals. It plays a causative role in chronic gastritis, peptic ulcer disease and is strongly associated with the development of gastric adenocarcinoma and mucosa-associated lymphoid tissue lymphoma [2]. The histopathological hallmarks of *H. pylori*-induced disease are a massive inflammatory cell infiltration of the lamina propria and erosion of the gastric epithelium. The gastric mucosa is well protected against bacterial infections. However, *H. pylori* is able to adapt and reside in the mucus, attach to epithelial cells, evade immune responses, and achieve persistent colonization in the stomach. It is not clear why the immune system fails to clear *H. pylori* infection. Furthermore, the mechanisms controlling the induction and maintenance of the *H. pylori*-induced chronic inflammation are only partly understood [3]. *H. pylori* is generally viewed as an extracellular pathogen colonizing the luminal side of the gastric epithelium and inducing an immune-inflammatory response on the stroma side of the mucosa. Paradoxically, *H. pylori*-specific gastric mucosal T cells generally present a Th 1 phenotype. It has been shown that Th1 cytokines promote gastritis, whereas Th2 cytokines are protective against gastric inflammation [4]. DCs are potent antigen-presenting cells that initiate T-cell responses. An abnormal pattern of DC maturation could promote chronic inflammatory processes [5]. Thus, the dysfunction of DCs may contribute to the local inflammation by producing inflammatory mediators and inappropriate activation of T cells.

Virulent *H. pylori* is capable of escaping phagocyte killing by delaying phagosome formation [6], [7]. However, we have reported the Taiwan clinical isolates such as HP238 and HP250, not only survive but also replicates in human macrophage cell line THP-1 cells [8]. In this study, we further extend this research to show that *H. pylori* can multiply in BMDCs, and impair the function of BMDC.

## Results

### Replication of *H. pylori* in murine BMDCs

**Figure 1:**
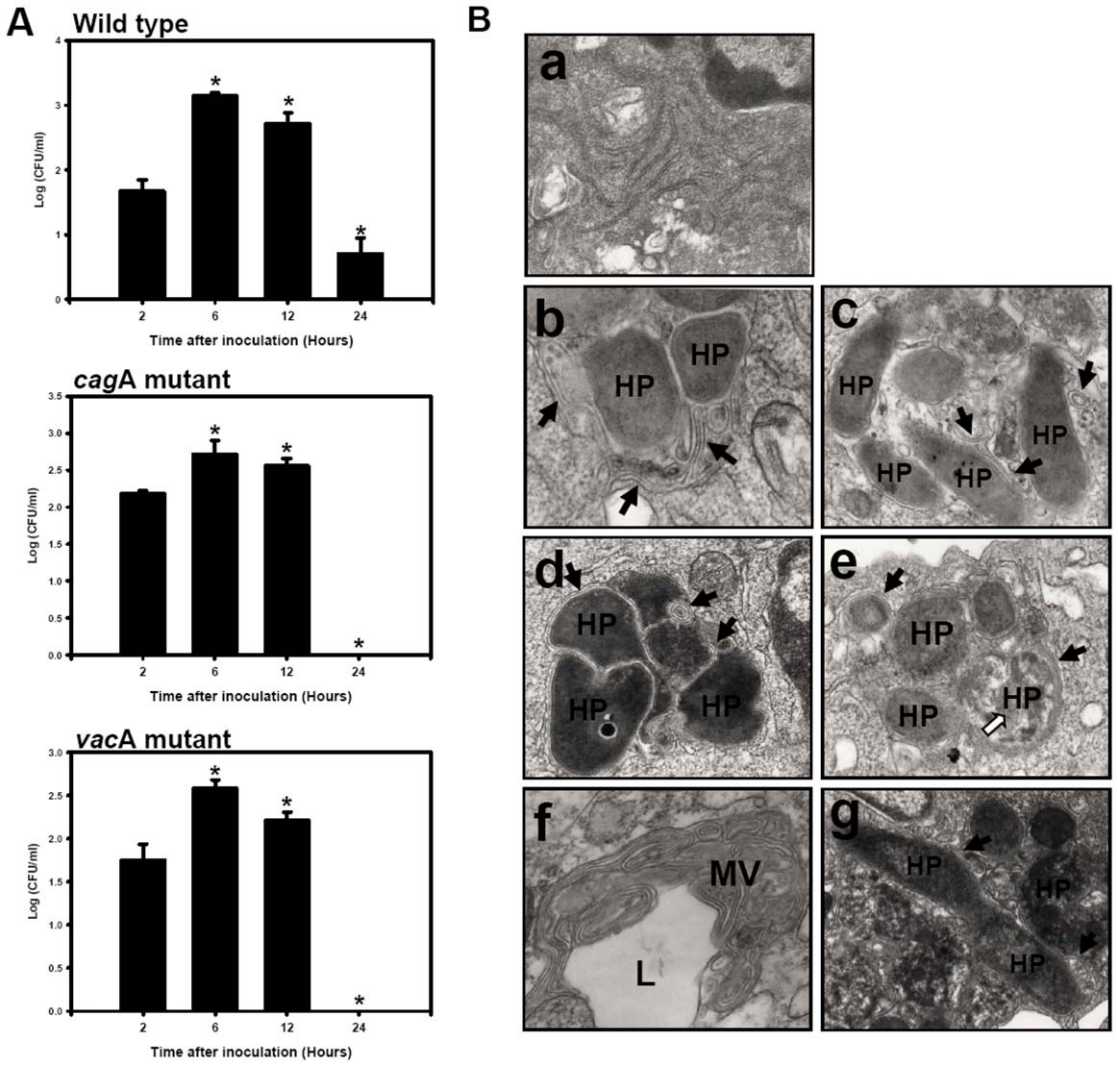
Multiplication of *H. pylori* in BMDC cells. (A) Multiplication of *H. pylori* in BMDCs. BMDCs infected with HP238 wild type, VacA or CagA mutants at m.o.i. = 10 for 2, 6, 12 and 24 h were lysed and intracellular bacteria were quantified at different time points after inoculation. The recovered viable *H. pylori* were determined as CFU on CDC plate. (B) Ultrastructural alterations in *H. pylori*-infected BMDCs. At 2 h (b, c), 6 h (d, g), 12 h (f), and 24 h (e) post infection, infected cells were collected for EM examination. (a) shows the mock samples without *H. pylori* infection. (g) shows the dividing bacteria at 6 h p.i. ‘HP’ indicates the location of *H. pylori* within BMDCs. Closed arrows indicate the double-layer structure whereas opened arrows indicate digested bacteria. (f) shows the multivesicular structure (MV) and lysosome (L) at 12 h p.i. * p<0.05 via student t-test.

*H. pylori* can multiply in macrophage autophagosomes [8]. To extend to DC, murine BMDC were infected with HP238 at a MOI of 10 for 1 h. Gentamycin was used to kill extracellular *H. pylori* for another 1 h. After gentamicine treatment, the cells were washed with PBS for 3 times and then cultured for additional 6, 12 and 24 h. At various time points post-infection (p.i.), BMDC were lysed, and viable *H. pylori* were re-cultured and plate-counted on a CDC plate for colony forming units (CFU) determination. As shown in Fig. 1A, CFU counts at 2 h p.i. corresponded to the amount of internalized live *H. pylori*. At 6 h p.i., a 10-fold increment was observed indicating that internalized *H. pylori* underwent replication. The number of live *H. pylori* gradually decreased at 12 h till 24 h p.i., suggesting that BMDCs gradually cleared the bacteria. The extracellular *H. pylori* was killed by the gentamicin, no viable *H. pylori* can be cultured from supernatant at 6, 12 and 24 h p.i. (data not shown). The role of VacA or CagA was also evaluated. The *cag*A and *vac*A mutants were also able to replicate in BMDC, but were cleared at a faster rate than wild type. No viable *H. pylori* could be recovered at 24 h p.i. (Fig. 1A).

### *H. pylori*-associated autophagy in infected murine BMDCs

**Figure 2:**
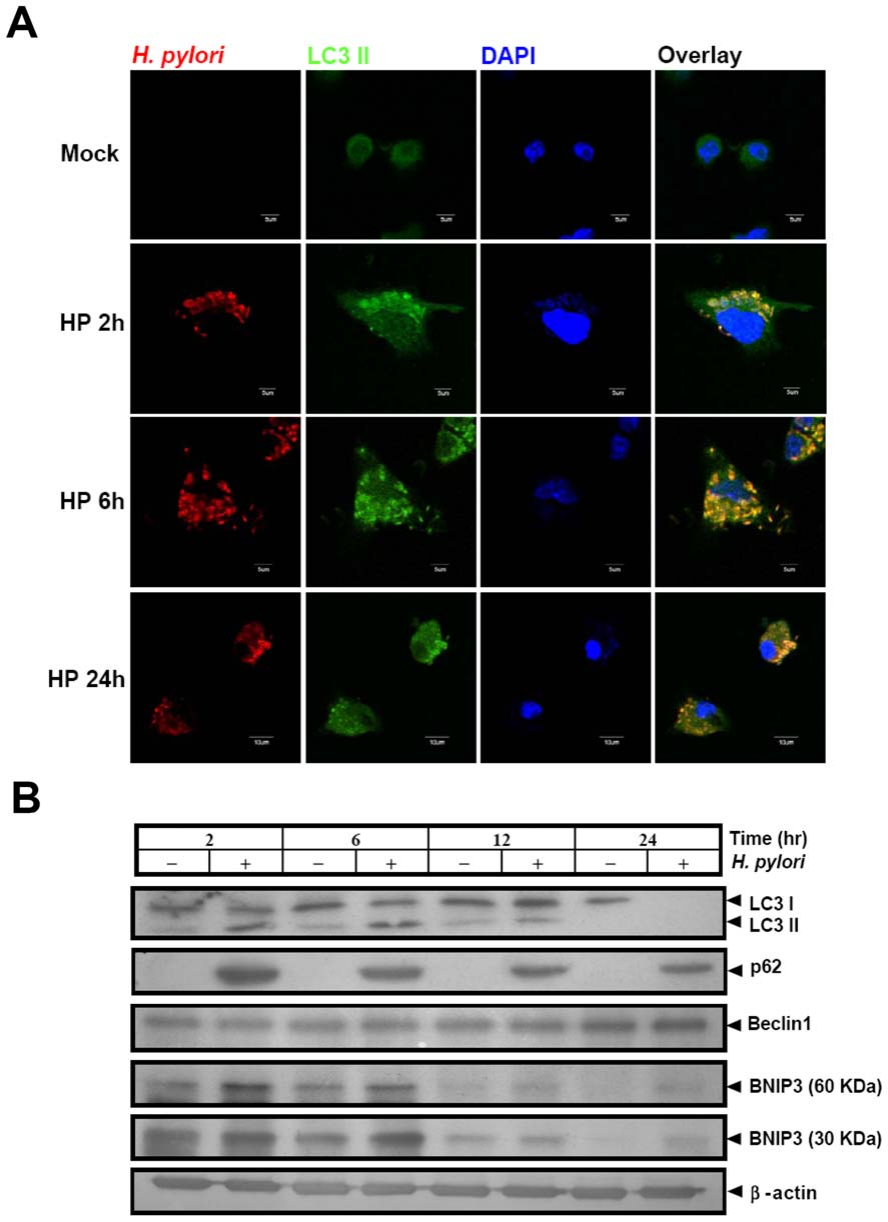
*H. pylori* infection in BMDCs induces autophagy. (A) BMDCs were infected with HP238 at m.o.i. = 10. At 2, 6, and 24 h post infection, the infected cells were stained with anti-*H. pylori* antibodies (red) and LC3-II antibodies (green). (B) LC3-II conversion and autophagic flux in *H. pylori*-infected BMDCs. BMDCs were infected with *H. pylori* at m.o.i. = 10 for 2, 6, 12 or 24 h post infection, protein samples were collected for LC3-II, p62, Beclin1, BNIP3 and β-actin analysis by Western blot.

Replication of *H. pylori* in murine BMDCs was examined under transmission electron microscopy. Intracellular *H. pylori* were found surrounded by a double layer membrane at 2, and 6 h p.i. (Fig. 1B-b,c), a characteristic of autophagosomes. Dividing *H. pylori* could also be observed in double-layer large membrane vacuoles (Fig. 1B-f). At 12 h p.i., several autophagosomes fused with lysosomes to form a onion-like structure of multiple layer vesicles (Fig. 1B-e) and bacteria degradation were observed at 24 h p.i. (Fig. 1B-d). The autophagic marker LC3-II co-localized with the *H. pylori*-containing vacuole at 2 h p.i. (Fig. 2A). Increased numbers and densities of LC3 punctate staining were associated with replicating *H. pylori* at 6 h p.i., but gradually decreased at 24 h p.i. On Western blot analysis, the signaling molecules such as LC3 II conversion, BNIP3 formation, but not in Beclin1, were also found to increase after infection compared to uninfected cells (Fig. 2B). The p62 was induced at 2 h p.i., and degrade gradually till 24 h, p.i., indicating that an autophagic flux was induced post infection. This suggests that *H. pylori* infection induces autophagy and autophagosomes seem to be the site in which *H. pylori* transiently replicate before degradation by autophagolysosome.

### Membrane trafficking of *H. pylori* and impairment of MHC class II molecule surface expression

**Figure 3:**
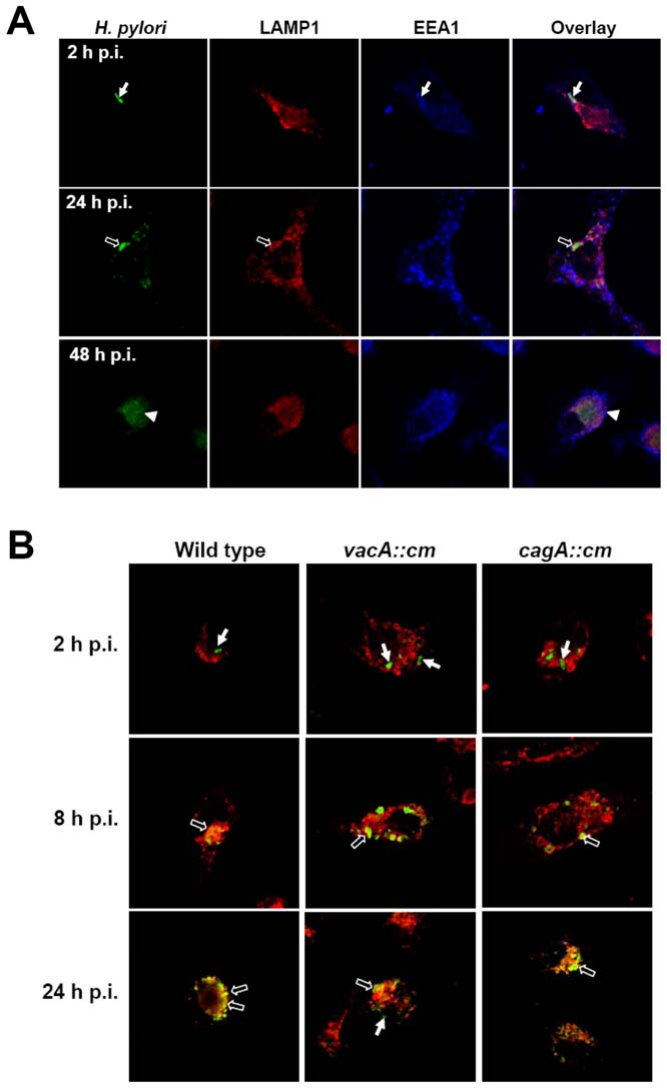
*H. pylori* in LAMP1^+^ vesicles within BMDCs. (A) The endocytic trafficking of *H. pylori* within BMDCs. BMDCs were infected with HP238 at m.o.i. = 10. At 2, 24, or 48 h post infection, the infected cells were stained with anti-*H. pylori* antibodies (green), anti-EEA1 antibody (blue), or anti-LAMP1 antibodies (red). Confocal images show the localization of *H. pylori* with EEA-1^+^ or LAMP-1^+^ vesicles. Closed arrows indicate EEA-1^+^ *H. pylori*-containing vesicles, opened arrows indicate LAMP-1^+^ *H. pylori*-containing vesicles and triangles indicate the digested bacteria particles. (B) *H. pylori* can replicate in LAMP1^+^ vesicles within BMDCs. BMDCs were infected with HP238 wild type, vacA (*vacA::cm*) and cagA (*cagA::cm*) mutant at m.o.i. = 10. At 2, 8 and 24 h post infection, the infected cells were stained with anti-*H. pylori* antibody (green) and anti-LAMP-1 antibodies (red). Confocal images show the localization of *H. pylori* on LAMP1^+^ vesicles. The closed arrows indicate the bacteria without LAMP-1 surround and the opened arrows show *H. pylori* ^+^ and LAMP-1^+^ vesicles.

A delayed phagosome maturation associated to the formation of megasomes was reported to delay the clearance of *H. pylori* in macrophages [7]. Therefore, *H. pylori* trafficking in murine BMDCs was studied by confocal microscopy. *H. pylori* was detected in EEA1 early endosome-positive vacuoles at 2 h p.i.. Then, *H. pylori* wild type, cagA and vacA mutants were found in a LAMP-1-positive compartment at 24 h p.i. (Fig. 3B). At 48 h p.i., loss in the intensity of anti-*H. pylori* antibody staining was observed indicating that *H. pylori* had started to be degraded (Fig. 3A).

**Figure 4:**
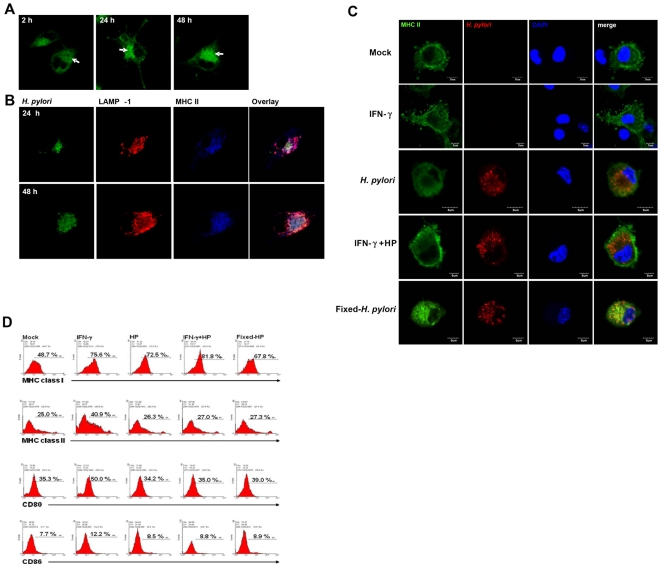
MHC class II cell surface expression is inhibited by *H. pylori*. BMDCs were infected with *H. pylori* at m.o.i. = 10. At 2, 24, or 48 h post infection, the infected cell was stained with anti-class II antibody only (A) or anti-*H. pylori* antibody (green), anti-LAMP-1 antibody (red), or anti-class II antibody (blue) (B). Confocal images (arrow) shows MHC class II expression on the infected BMDCs. Arrows show the MHC class II accumulated in cytoplasm (A). Confocal images show the co-localization of *H. pylori*, LAMP-1 and MHC class II molecules on infected BMDCs at 24 and 48 h post infection (B). (C) *H. pylori*-infected cells were stained with anti-class II antibody (green) and anti-*H. pylori* antibodies (red), and DAPI (blue). Confocal images shows MHC co-localization of class II and *H. pylori*-containing vesicle on various treatment groups (mock control, IFN-γ treatment, *H. pylori* infected, *H. pylori* with IFN-γ or formalin fixed-*H. pylori*) at 24 h post treatment. (D) Surface class II expression on *H. pylori*-infected BMDCs. *H. pylori*-infected cells were surface stained with anti-class I, class II, CD80, or CD86 antibody, and analyzed with Flow cytometry. The percentage of stained antibody positive cells is depicted.

DCs are the most potent antigen presenting cells. Activated DCs express activation molecules, such as CD80 and CD86 and MHC class II molecules on the cell surface to present antigenic peptides to activate T cells. In *H. pylori*-infected murine BMDCs, MHC class II molecules were not transported to the cell surface and accumulated in the cytoplasm (Fig. 4A). In fact, MHC molecules were found to localize with the *H. pylori*-containing vacuoles (Fig. 4B). Under these conditions, *H. pylori* infection also impaired the IFN-γ -induced up-regulation of MHC class II expression. MHC class II molecule transport to cell surface was also defective in formalin-fixed *H. pylori* infected BMDCs. Since the formalin-fixed *H. pylori* is not capable of replicating, this suggests that *H. pylori* replication is not a prerequisite for the block of class II molecule transport to cell membrane (Fig. 4C). We used flow cytometry to stain the surface expression of MHC class I, II molecules and CD80 and CD86 in *H. pylori*-infected BMDC. The IFN-γ was used as a positive control to enhance the surface expression of MHC class I and II molecules, CD80 and CD86. MHC class I expression was enhanced after *H. pylori* infection and the addition of IFN-γ further increased MHC class I expression, indicating that *H. pylori* indeed activated MHC class I expression in BMDC. In contrast, MHC class II, CD80 and CD86 surface expression remained at the same level as untreated control immature cells (Fig. 4D). *H. pylori* infection also down-regulated the IFN-γ enhanced MHC class II, CD80 and CD86 surface expression. Interestingly, formalin-fixed *H. pylori* infection induced the same activities as untreated bacteria. Therefore, transport of MHC class II molecules from the cytoplasm to the plasma membrane seems to experience interference from the presence of the *H. pylori*, either viable or formalin-fixed.

### *H. pylori* infection stimulates murine BMDCs to secrete TNF-α, IL-6 and IL-10, but not IL-12

**Figure 5:**
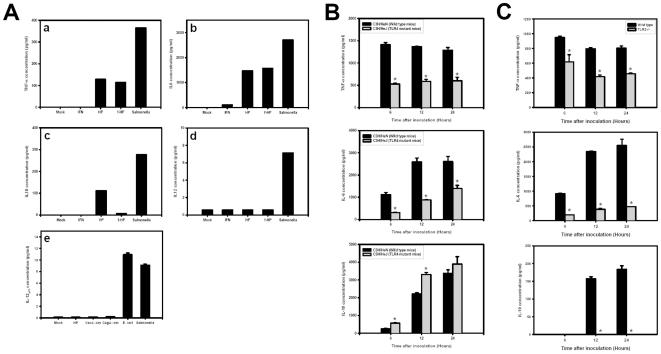
TNF-α, IL-6, IL10 and IL-12_p70_ production of *H. pylori*-infected BMDCs. Cytokine production in *H. pylori*-infected BMDCs derived from B6 mice (A), TLR4 deficient mice (B), or TLR2 knock out mice (C). BMDCs were infected with *H. pylori* at m.o.i. = 10. At 24 h post infection, the supernatants were collected from medium alone (mock), *H. pylori*, formalin fixed-*H. pylori* or *Salmonella* treated BMDCs and the TNF-α (a), IL-6 (b), IL-10 (c), and IL-12_p70_ (d,e) concentrations were determined. In (e), the vacA (VacA::cm), cagA (CagA::cm) mutants were included for comparison. (B &C) TNF-α, IL-6, and IL-10 production in *H. pylori*-infected BMDCs derived from wild type, TLR4 deficient, or TLR2 knock out mice, the cytokines concentration were determined at various hour post infection. * p<0.05 via student t-test.

The cytokine secretion pattern after *H. pylori* infection was further analyzed at 24 h p.i.. *Salmonella* enteric serovar Thyphimurium infection was used as a positive control. TNF-α and IL-6 secretion in supernatants from *H. pylori* or formalin-fixed *H. pylori* infected cells were found to be similar (Fig. 5A). Interestingly, IL-10 secretion required BMDCs stimulated by live *H. pylori* only. No IL-12 secretion was observed from the *H. pylori*-infected BMDCs (Fig. 5A-d). The *vacA* and *cagA* mutants were also tested for the production of IL-12. These mutants also failed to produce IL-12 (Fig. 5A-e). These results suggest that *H. pylori* infection impairs the activation and maturation of DCs, probably by down regulating the secretion of pro-iflammatory cytokines such as IL-12 and, in the case of live bacteria by promoting the secretion of IL-10.

### Negative role of TLR4 or TLR2 on the BMDC maturation post *H. pylori*-infection

**Figure 6:**
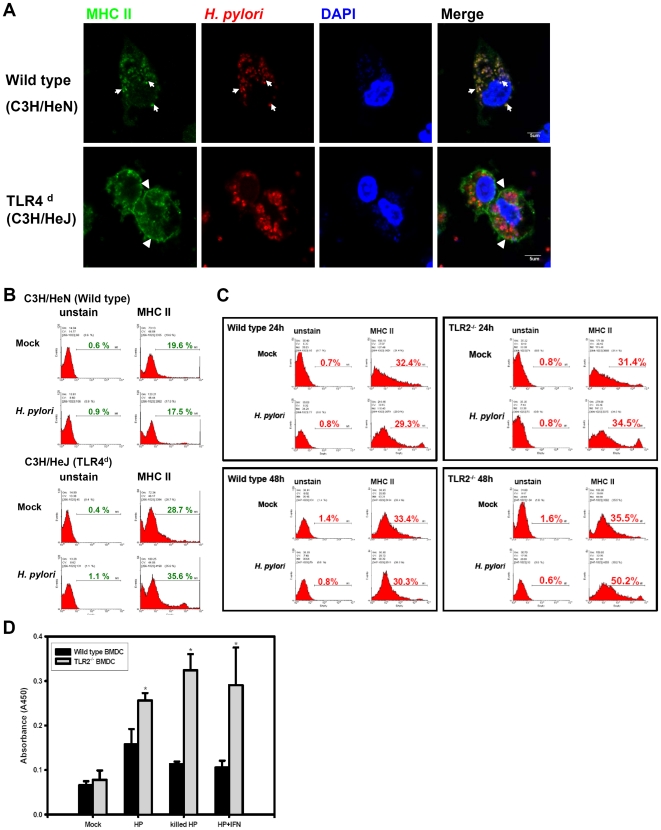
TLR 2 or TLR4 negatively regulate *H. pylori*-mediated inhibition of MHC class II surface expression and antigen presentation. BMDCs derived from TLR4 deficient C3H/HeJ (A & B) or TLR2 knock-out B6 mice (C) were infected with *H. pylori* at m.o.i. = 10. The MHC class II expression was detected on confocal microscopy (A) or flow cytometry (B) at 24 h post inoculation. In A & B, the wild type (C3H/HeN) was compared to the TLR 4 deficient (C3H/HeJ) mice. In C, the B6 wild type was compared to the TLR2 knock out mice. (D) Antigen-specific T cell proliferation. BMDCs derived from B6 or TLR2^-/-^ mice were infected with *H. pylori*, heat-killed *H. pylori*, or IFN-γ plus *H. pylori* for 24 h. Then BMDCs were mixed with *H. pylori*-primed lymph node cells at a 1∶5 ratio. The T cell proliferation was determined after 5 days by BrdU cell proliferation ELISA kit. The absorbance at 450 nm was depicted. Wild type (▪) and TLR2^-/-^ (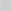) BMDCs were compared for their antigen presentation to *H. pylori*-specific T cells. *H. pylori*-stimulated wild type BMDCs have lower antigen presentation than that of TLR2^-/-^. * p<0.05 via student t-test.

*H. pylori* LPS uses both TLR4 and TLR2 for cell activation [9]. Hence, we compared *H. pylori* infection in BMDCs derived from TLR4-deficient C3H/HeJ mice and in C3H/HeN control mice. Although the presence of *H. pylori* in BMDC from TLR4-deficient C3H/HeJ mice is slightly lower than that from C3H/HeN mice (Figure S1A), *H. pylori* can replicate in TLR4-deficient BMDC. However, the MHC class II surface expression in infected TLR4-deficient BMDCs is not inhibited (Fig. 6B) and no co-localization of MHC class II molecules with *H. pylori*-containing vesicles was found (Fig. 6A). For cytokine production, TNF-α and IL-6 secretion were lower in infected TLR4-deficient BMDC than in wild type BMDC. However, IL-10 secretion was higher in TLR4-deficient BMDC than in wild type BMDC (Fig. 5B). No IL-12 production was detected for both BMDCs (data not shown).

We also compared DC activities in wild type versus TLR2 knockout mice. *H. pylori* can replicate in BMDCs derived from either TLR2 wild type or TLR2^-/-^ (Figure S1B). In TLR2^-/-^ BMDC, MHC class II surface expression inhibition was transient, found only at 24 h p.i. Then, an enhanced surface expression of MHC class II molecules was observed at 48 h p.i.(Fig. 6C). The cytokine expression was also modulated in TLR2^-/-^. TNF-α and IL-6 were lower in TLR2^-/-^ BMDC than in wild type BMDC. However, in contrast to what is observed in TLR4-deficient mice, IL-10 was not detected in the supernatants of TLR2^-/-^ infected BMDC (Fig. 5C).

Finally, we assayed antigen presentation in *H. pylori*-infected BMDC to *H. pylori*-specific T cells. Lymph node T cells derived from *H. pylori*-immunized mice were stimulated with *H. pylori*-infected BMDC in a ratio of 1∶5 and the BrdU incorporation was determined. Comparing with the *H. pylori*-infected TLR2^-/-^ BMDC that has an increased MHC class II molecule surface expression post-infection, *H. pylori*-induced T cell proliferation by wild type BMDC was significantly lower than that of TLR2^-/-^ BMDC (Fig. 6D). Whether *H. pylori* infected BMDC, killed *H. pylori*-treated BMDC, or IFN-γ treated *H. pylori*-infected BMDC, the same pattern was displayed, indicating that *H. pylori*-treated BMDC showed a defect in antigen presentation.

## Discussion

We have demonstrated here that *H. pylori* is capable of multiplying in BMDCs after invasion. A 10-fold increase of CFU between 2 h and 6 h p.i. is found in *H. pylori*-infected BMDCs. The doubling time of the *H. pylori* in BMDC cells was calculated to be 1.5–2 h. Autophagy is induced by *H. pylori* and the double-membrane autophagosomes is associated *H. pylori* after its invasion. Then, the *H. pylori*-containing autophagosomes fuse with lysosomes to lead to degradation of the bacteria. The finding of this transient replication of *H. pylori* in BMDCs suggests that *H. pylori* can be considered as a facultative intracellular microorganism under certain condition. Moreover, its multiplication in BMDCs would impair the functions of the dendritic cells.

*H. pylori* is generally viewed as a non-invasive pathogen localized only in the lumen of the stomach and attached to gastric epithelial cells. It has adapted several mechanisms to reside and persist in the mucus, avoid phagocytosis and evade immune responses. The phagocytosis process is retarded and the membrane trafficking of phagosome maturation is disrupted to form a megasome that contains the ingested bacteria [6], [7]. Many reports have shown that *H. pylori* is invasive, and can be considered a facultative intracellular organism [10], [11]. However, no direct demonstration of its replication by plaque counting in infected cells has so far been provided. We have found that *H. pylori* could multiply not only in macrophages [8], and in epithelial cells (data not shown), but also in BMDCs as shown in this study. Two Taiwanese clinical isolates of HP238 (from gastric adenocarcinoma specimen) and HP250 (from mucosa-associated lymphoid-tissue lymphoma speciment) can multiply in macrophage cell line THP-1 [8] whereas standard strains of ATCC43504 and J99 can multiply in AGS or MKN45 epithelial cell line (unpublished data). For BMDCs, only HP238 is found to replicate in this study. This new finding has several implications for the biological life cycle of *H. pylori* in the host, especially considering the role of DC as controllers of the immune responses. Wunder *et al*. reported that *H. pylori* is auxotrophic for cholesterol, and can extract cholesterol from epithelial membrane. The glycosylation of cholesterol would inhibit the phagocytosis of *H. pylori* and promote its immune evasion [12]. Cholesterol has been found to play an essential role in the establishment of the chronic infection of intracellular microorganisms, in that the phagosomal lipid metabolism of the host macrophage was modulated by the virulent factors of the bacteria to favor the survival of the invading bacteria [13]–[15]. *H. pylori* can also infect the AGS cells via a sipper-like mechanism, involving intimate contact with AGS cells microvilli and surface membrane pseudopod structure. The internalized *H. pylori* are found in LAMP-1-containing vacuoles [16]. Cytoskeletal rearrangement with tyrosine phosphorylation was observed in the promixity of intracellular *H. pylori*. We found that *H. pylori* infection induced autophagosome formation in either phagocytic THP-1 cells, BMDC, or non-phagocytic AGS epithelial cells. *H. pylori* resides and replicates within autophagosomes, probably reflecting its dependence on the cell membrane probably because of its cholesterol requirement for growth. The CagA can be translocated to plasma membrane and form rafts with the invading *H. pylori* [17]. In our study, Cag A or VacA mutants can still replicate in BMDC, but were cleared in a fast rate probably due to the interference in the autophagosome formation by Cag A or VacA. This needs further investigation. Rittig et al reported the *H. pylori*-induced homotypic phagosome fusion is independent of the VacA or Cag A in human monocytes [18]. Since we used murine BMDC, whether *H. pylori* can replicate in human dendritic cells need to be checked in the future.

In view of the observation that *H. pylori* infection induces autophagy, there remains the question as to what role autophagy plays in the defense against *H. pylori*. Autophagy has been found to be a component of the innate cellular immune responses against not only intracellular but also against extracellular microorganisms [19], [20]. After phagocytosis of the microorganism, the autophagosomes will be formed to degrade the ingested bacterium by the lysosomal killing mechanism. The intracellular bacteria such as *Legionella pneumophila, Listeria monocytogenes*, *Mycobacterium*, and *Shigella* have developed different mechanisms to evade the autophagic cellular surveillance in macrophage. The double membrane-bound compartments that bear autophagic markers were induced, but its maturation into autophagolysosomes will be arrested or delayed. Allen *et al*. have reported the megasome formation induced by *H. pylori* via homotypic phagosome fusion to delay its clearance in macrophage [6], [7]. However, for the *H. pylori*-induced autophagosome, the autophagosome not only provides the site for replication, but also the place for the destruction of the replicating bacterium. After phagocytosis into BMDC, *H. pylori* co-localized with EEA1 early endosomal marker and then with the LAMP1^+^ late endosomal/lysosomal marker (Fig. 3). Multiple vesicle vacuoles formed at 12–24 h p.i. that subsequently resulted in the destruction of the bacteria due to fusion with lysosomes (Fig. 1B). IFN-γ treatment enhanced the clearance of the *H. pylori* also supporting the notion that autophagy helps the clearance of internalized *H. pylori* in the endosome/autophagosome after fusion with lysosomes (Figure S2).

TLRs are involved in the innate immune response to *H. pylori* infection [9], [21]–[23]. *H. pylori* has adapted its LPS structure for colonization, adhesion and escaping from immune responses. The phase-variable expression of these Lewis antigens seems to affect the induced immune response by modulation of cell activation through DC-SIGN. Le^X^ antigen negative expressed *H. pylori* escape binding to dendritic cells and induce a strong Th1 response. In contrast with Le^X^ antigen negative expression, *H. pylori* variants that express Le^X^/Le^Y^ can bind to DC-SIGN on dendritic cells and enhance the production of IL-10 which promote Th2 response [24], [25]. Cell activation by *H. pylori* LPS has been shown to be both TLR2- and TLR4-dependent, although less stimulatory than *E. coli* LPS, and sometime antagonistic for TLR4 [26]–[28]. For *H. pylori* LPS, TLR2 is primarily activated [21], [28], and induces the production of IL-10, also skewing the immune response to Th2 arm [9], [25], [29], [30]. In our study, the secretion of TNFα and IL-6 was inhibited in TLR4- and TLR2-deficient BMDCs, indicating that both TLR2 and TLR4 participate in the response to *H. pylori* LPS. Intriguingly, IL-10 production was enhanced in TLR4-deficient BMDCs, but completely abolished in TLR2^-/-^ BMDCs. TLR4 was reported to negatively regulate the fusion between phagosomes and lysosomes in macrophage for the degradation of the engulfed antigen [31]. IL-10 has been found to down-regulate the MHC class II export to plasma membrane, probably via inhibition of cathepsin S [32], [33]. How the *H. pylori* components interfere with the MHC class II molecules transport to cell surface is intriguing. VacA is initially characterized as its ability to induce the formation of intracellular vacuoles in cell lines, and has been found to decrease the pH value of acidic vesicle. Molinari M., *et al*., demostrated VacA interferes the Ii-dependent pathway of antigen presentation in B cells [34]. During the phagosome maturation, the VacA-containing *H. pylori* or VacA recruit and retain the coronin 1 to disrupt the phagosome maturation and inhibit the antigen presentation [34], [35]. The urease of *H. pylori* can also bind to CD74 (the class II-associated invariant chain that regulate the intracellular transport and functions of class II) to affect the cell motility of dendritic cells [36], or cause megasome formation [37]. In addition, several molecules such as RP105, ST2, SIGIRR, or Galectin-3 have been reported to regulate the TLR4 or TLR2 signaling negatively [38]–[43]. In our study, surface MHC class II molecules, or even after IFN-γ-treatment, were down-regulated by *H. pylori* infection in BMDCs. The formalin-fixed and heat-killed *H. pylori* can still remain the inhibition of the MHC class II expression. The MHC class II molecule seems to be trapped in *H. pylori* containing vesicles. The TLR-2 and 4 signaling also play a negative role on the class II surface expression. It must be a multiple factors involvement. The Ii, urease, IL-10, TLR2/4 signaling will all contribute to the inhibition of class II export and these are under current investigation.

DC can phagocytose and process bacteria for antigenic peptide presentation by MHC class II molecules to specific T cells. DCs are activated and secrete IL-12, which favors the differentiation of splenocytes into Th1 [4], [27], [44]–[46]. Inhibition of IL-12 secretion by *H. pylori* was also reported [47], [48]. In this study, IL-10, but not IL-12, was detected after *H. pylori* infection. In addition, MHC class II molecule export to the cell surface is blocked, which results in an impaired antigen presentation. This will favor the induction of a non-Th1 response. A complexity of interaction between *H. pylori* and host immune defenses is responsible for the persistence of *H. pylori* infection [3]. For *H. pylori*, Th1 has been supposed to be protective as well as immunopathogenic, which is paradoxical. CD8^+^ T cells are also activated and are associated with severe gastritis in *H. pylori*-infected mice [49]. The replication of *H. pylori* within the cells can explain the endogenous antigen presentation and activation of CD8^+^ T differentiation post infection. In conclusion, the finding that *H. pylori* can multiply in dendritic cells will add a new element to the complexity of the immune responses to the *H. pylori* infection, and shed light on further understanding of immunopathology in *H. pylori*-induced disease.

## Materials and Methods

### Bacterial strains and culture

The *H. pylori* clinical isolate (HP238) was obtained from the Department of Pathology, National Cheng Kung University Hospital. The mutant of *cag*A and *vac*A were used as previously described [8]. *H. pylori* were grown on CDC anaerobic 5% sheep blood agar plates (BBL, Becton-Dickinson, USA) under microaerophilic conditions (5% O_2_, 10% CO_2_, 85% N_2_) and 85% humidity in a Nuaire incubator (Plymonth, Minnesota, USA) at 37°C. Fresh plates were started from glycerol stocks and subculture every 48 hours. In bacterial replication assays, the strain used in this study were HP238, and its *vacA* or *cagA* isogenic mutants [8] at a multiplicity of infection of 1∶10. Bacteria were centrifuged onto BMDCs at 400 g for 6 min at 4°C and incubated at 37°C with 5% CO2 atmosphere one hour. After washed with phosphate-buffer saline (PBS) for 3 times, the cells were incubated with 5% FBS, 100 µg/ml gentamicin RPMI1640 media at 37°C for another one hour. After gentamicine treatment, the cells were washed with PBS for 3 times and then cultured with dendritic cell culture media for 6, 12 and 24 h. After co-incubation of BMDC with H. pylori for 6, 12 or 24 h, BMDC were lysed with 0.5 ml of 0.1% saponine (in DPBS) and then plated on CDC plates with serial dilution to determine the viable bacteria. Colonies were grown and counted after 7 days culture. The time point of 2 h is considered as the entry of *H. pylori* into the cells. The increase of CFU at 6 h is interpreted as result from multiplication of *H. pylori* within the cells. There was no viable bacterial cultured from supernatant post 6, 12 and 24 h post infection (p.i.). Bacteria were treated with 4% formalin for 10 min and then washed 3 times with PBS. *H. pylori* was heat-killed at 95°C for 20 min.

### Bone marrow-derived dendritic cells culture

Bone marrow cells were collected from the bones of 6-10-week old C57BL/6 (wild and TLR2 knockout) mice, C3H/HeN or C3H/HeJ female mice. The 1.6×106/well cells were cultured in 6-well plate with RPMI1640 media containing 10% fetal bovine serum, 50 µM 2-mercaptoethanol and 20 ng/ml mouse recombinant GM-CSF at 37°C for 5 days to differentiate into dendritic cells. The media were replaced with fresh media on 3rd day. The mice were maintained in the pathogen-free facility of the Animal Laboratory of National Cheng Kung University (Tainan, Taiwan) that were raised and cared for according to the guidelines set up by the National Science Council, ROC. The use of mouse experiments were approved by the institutional animal care and use committee of NCKU.

### Flow cytometry

BMDC were infected with *H. pylori* at m.o.i. = 10 at 37°C for 1 h and then washed and treated with 100 µg/ml gentamicin. The infected cells were harvested at different times post infection, and were stained with anti-class I, class II, CD80, and CD86 antibody (eBioscience, USA) on ice for 30 min and then washed with wash buffer. The percentage of antibody positive cells was analyzed by the FACSCalibur flow cytometry system.

### Immunofluorescence confocal microscopy

BMDC were incubated with either *H. pylori*, or formalin-fixed *H. pylori* for 1 h, then washed and treated with 100 µg/ml gentamicin. For the IFN-γ treated groups, recombinant mouse IFN-γ (100 U/ml) was added after the gentamicin treatment step, and were then fixed with 3% paraformaldehyde, pH 7.4, at room temperature for 10 min, and washed with phosphate buffer saline 3 times. The primary antibodies used were: rabbit anti-*H. pylori* antibody (ABR, USA), mouse anti-*H. pylori* (Abcam, UK), rabbit anti-LC3 II antibody (Abgent, USA); rat anti-lysosomal-associated membrane glycoprotein 1 (LAMP-1) antibody 1D4B (eBioscience, USA); goat anti-human EEA-1 antibody (Santa Cruz, USA), and rabbit anti-MHC class II antibody. The secondary antibodies used were: FITC-conjugated goat anti-rabbit, TexRed-conjugated donkey anti-rat and cyanin-5-conjugated anti-goat antibodies. Samples were observed on a Zeiss LSM 510 laser scanning confocal microscope for image acquisition.

### Cytokine detection

Supernatants were collected from medium alone (mock) or bacterial infected groups after inoculation. TNF-α, IL-6, IL-10, and IL-12_p70_ concentrations were detected by ELISA kit (R&D, USA).

### Western blot analysis

The cells were harvested at different times post infection, and lysed in lysis buffer (25 mM Tris, 137 mM NaCl, 10% (v/v) glycerol, 0.5% (w/v) sodium deoxycholate, 2 mM EDTA) containing protease inhibitors (1 mM phenylmethylsulfonyl fluoride, 30 µg/ml aprotonin, 1 mM sodium orthovanadate). Lysates were clarified by pipetting and vortex at 4°C, 30 min. Insoluble material was removed by 12,000 rpm centrifuge. Protein concentrations were determinate by Bio-Rad protein detection kit. Equal amounts of protein were loaded on a 12% SDS-PAGE, transferred to nitrocellulose membrane. Anti-Beclin 1 (Santa Cruz, CA), BNIP3 (Sigma, USA), p62 (Abgent, USA), LC3 II (Abgent, USA) and GAPDH (Abcam, Cambridge, UK) antibodies were used to detect protein expression patterns. After incubation with peroxidase-conjugated secondary antibodies, the blots were visualized by enhancing chemiluminescence reagents (Perkin Elmer Life Sciences, Boston, MA).

### Transmission electron microscopic examination

For ultrastructural analysis, *H. pylori* infected cells at different time points were fixed with 4% glutaraldehyde and post-fixed in 1% OsO_4_. The cells were observed under transmission electron microscopy (Hitachi 7000, Japan).

### T cell proliferation

To sensitize the *H. pylori*-specific lymphocyte population, 6-week old female C57BL/6 mice were immunized with complete Freund's adjuvant and 1×10^8^ *H. pylori* 1∶1 (vol: vol) mixture at mouse footpads. At 5–6 days, lymphoid nodes were collected from immunized mice and lymphocytes were used to co-culture with PBS (mock), *H. pylori*-infected, heat-killed treated or IFN-γ + *H. pylori*-infected BMDC at 5∶1 for 5 days in 96-well plate. Lymphocyte proliferation was performed with 5-Bromo-2'-deoxy-uridine Labeling and Detection Kit III (Roche, USA).

## Supporting Information

Figure S1 Multiplication of *H. pylori* in BMDCs is TLR2 and 4 independent. The BMDCs derived from TLR4 deficient (A) or TLR2 knock-out (B) mice were infected with *H. pylori* at m.o.i. = 10. The recovered viable *H. pylori* were determined as CFU on CDC plate at 2, 6, 12 and 24 h post infection. Wild type (black) and TLR mutant (gray) BMDCs were compared for their support of bacterial replication.
(0.25 MB TIF)

Figure S2 IFN-gamma enhances the elimination of *H. pylori* in BMDCs. BMDCs were infected with *H. pylori* at m.o.i. = 10 for 1 h, and the IFN-gamma (100 IU/ml) was added after the gentamicin treatment step. The recovered viable *H. pylori* were determined as CFU on CDC plates at 2, 6, 12 and 24 h post infection. * p<0.05 via student t-test.
(0.14 MB TIF)
