# Supplementary figures and images for: Helicobacter pylori Impairs Murine Dendritic Cell Responses to Infection
Source: PLoS One. 2010 May 27;5(5):e10844. doi: 10.1371/journal.pone.0010844 (PMC2877707; doi:10.1371/journal.pone.0010844)

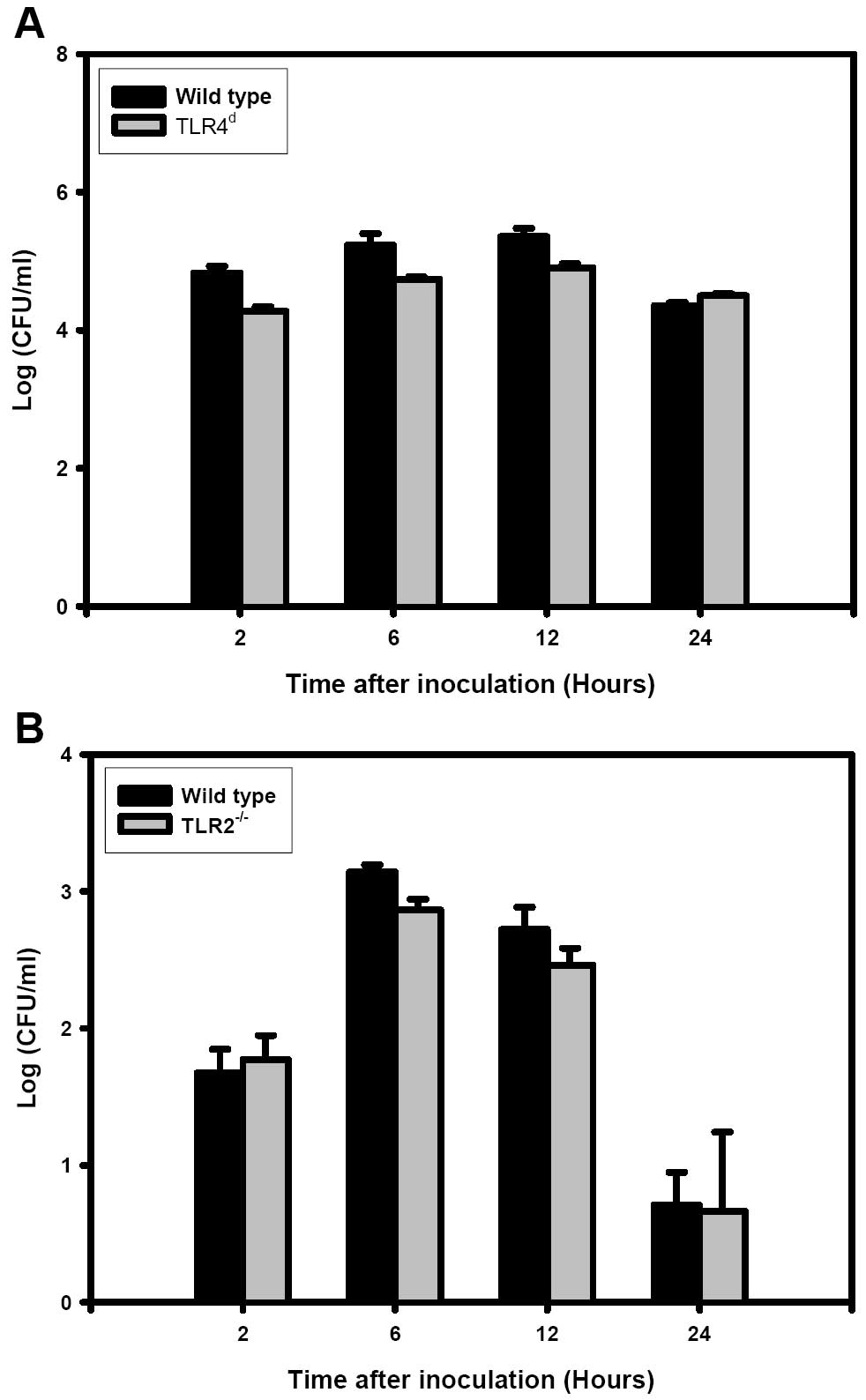

Supplement: Figure S1 — Multiplication of H. pylori in BMDCs is TLR2 and 4 independent. The BMDCs derived from TLR4 deficient (A) or TLR2 knock-out (B) mice were infected with H. pylori at m.o.i. = 10. The recovered viable H. pylori were determined as CFU on CDC plate at 2, 6, 12 and 24 h post infection. Wild type (black) and TLR mutant (gray) BMDCs were compared for their support of bacterial replication. (0.25 MB TIF) [file pone.0010844.s001.tif]

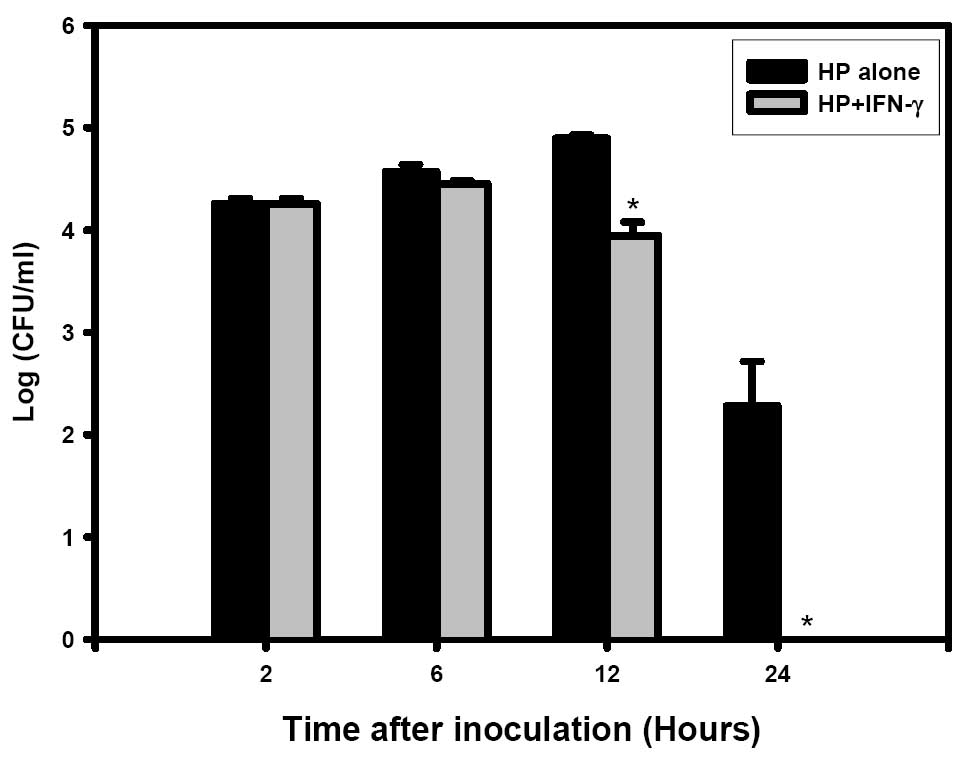

Supplement: Figure S2 — IFN-gamma enhances the elimination of H. pylori in BMDCs. BMDCs were infected with H. pylori at m.o.i. = 10 for 1 h, and the IFN-gamma (100 IU/ml) was added after the gentamicin treatment step. The recovered viable H. pylori were determined as CFU on CDC plates at 2, 6, 12 and 24 h post infection. * p<0.05 via student t-test. (0.14 MB TIF) [file pone.0010844.s002.tif]
